# Using different levels of energy and protein and their effects on bodyweight and blood chemistry of ostriches

**DOI:** 10.1007/s11259-021-09792-5

**Published:** 2021-04-28

**Authors:** Tahereh Nikravesh-Masouleh, Alireza Seidavi, Magdalena Solka, Mohammad Dadashbeiki

**Affiliations:** 1 Department of Animal Science, Rasht Branch, Islamic Azad University, Guilan 413353516 Rasht, Iran; 2 Department of Genomics and Biodiversity, Institute of Genetics and Animal Biotechnology of the Polish Academy of Sciences, 05-552 Jastrzębiec, Magdalenka, Poland; 3 Department of Veterinary Medicine, Rasht Branch, Islamic Azad University, Guilan 413353516 Rasht, Iran

**Keywords:** Energy, Protein, Blood biochemistry, Bodyweight, Ostrich

## Abstract

To determine the effect of different dietary energy and protein levels on bodyweight and blood chemistry, 36 ostriches at 2 to 9 weeks of age for feeding conditions and 18 for blood chemistry parameters was used. The birds were divided into six treatment groups. Energy and protein levels of diet were 2400 and 2600 kcal/kg and 20%, 22%, and 24%, respectively. The feed intake and bodyweight gain were determined a weekly. Blood chemical parameters including glucose, HDL, LDL, total cholesterol, triglycerides, total protein, albumin, globulin, aspartate amino-transferase and alanine amino-transferase activity were determined. The highest weight gain during the whole experiment was observed in ostriches offered 2400 kcal · kg^−1^ dietary energy and 20% protein. The lowest level of total cholesterol and protein was observed in treatment V (2600 kcal · kg^−1^ dietary energy and 22% protein). The lowest level of glucose and triglycerides was noted after treatment I. The highest albumin and globulin concentrations were in treatment III (2400 kcal · kg^−1^ dietary energy and 24% protein) and treatment II (2400 kcal · kg^−1^ dietary energy and 22% protein), respectively. The energy level had no effect (*P* < 0.05) on feed intake and weight gain in all experimental period. The results of this study showed that with increasing energy and protein levels, most blood parameters increased in ostriches but total cholesterol did not.

## Introduction

There is increased interest in rearing ostriches commercially in many countries of the world because of good adaptation of these birds to the environmental conditions (Cloete et al. 2012). Many valuable products are obtained from ostrich, e.g., meat, eggs, skin and feathers, which are important for different branches of production (Cooper 2004; Cloete et al. 2012). Therefore, the demand for information about this bird, especially its maintenance, nutritional requirements and the potential for genetic improvement has increased (Horbańczuk et al. 2007; Kawka et al. 2007). Several studies have determined the genetic structure of the ostrich, genetic variation among the breeds and dependence between populations (Kawka et al. 2010, 2012a, b).

However, nutrition is very important in ostrich farming, because it accounts for about 75% of the total production costs (Jordaan et al. 2008). Most of the ostrich performance problems relating to fertility can be traced back to poor breeder diet (Cooper et al. 2005). The most important feed ingredients are protein and energy. While there are some excellent papers on ostrich nutrition (e.g., Cloete et al. 2012; Jouki et al. 2014a, b; Poławska et al. 2014), the our knowledge is not enough as compared to other species of poultry.

In global literature, a few publications can be found that relate to feeding of ostriches based on feed of different origin and concentration of energy (Cooper and Horbańczuk 2004; Viljoen 2011; Brand et al. 2017; Karimi-Kivi et al. 2014, 2015; Nikraesh-Masouleh et al. 2018; Tasirnafas et al. 2014, 2015). However, research on the performance of commercial ostriches fed diets differing in protein and dietary energy is still lacking. Therefore, the aim of the present study was to determine the effect of two different levels of metabolizable energy (2400 and 2600 kcal/kg) and three different levels of protein (20, 22 and 24%) on the bodyweight and blood parameters of ostriches.

## Materials and methods

### Animals

This research was conducted on an ostrich farm located in Guilan, Iran, when the birds were between 2 and 9 weeks of age. For the first month after hatching, the ostriches were bred for growth and adaptation. After this period, 36 ostriches (*Struthio camelus*), both blue and black breed with equal weight, were selected. The selected ostriches were housed in 5 × 3 m land cages (two ostriches /cage). Temperature, humidity, lighting, health programs and other management factors were applied based on standard protocols. The ostriches were vaccinated following the standard vaccination schedule. Briefly, the ostriches were vaccinated against Newcastle (14th and 28th days of age), influenza (14th and 28th days of age), and Enterotoxemia (35th day of age). The experimental protocol (No. 911130) was authorized by the Institutional Animal Care and Ethics Committee of Islamic Azad University, Rasht Branch, Rasht, Iran.

## Treatments, experimental diets and measured traits

The 36 ostriches were allocated to six treatments. Treatments from I to III were given a diet with 2400 kcal · kg^−1^ of metabolizable energy and with 20%, 22%, and 24% of dietary protein. Treatments from IV to VI were given a diet of 2600 kcal · kg^−1^ metabolizable energy with 20%, 22% and 24% of dietary protein. The ingredients and composition of the diets are shown in the Tables 1 and 2, respectively. Food and drinking water were offered *ad libitum*.

**Table 1:** Selected feed ingredients of used diets during experiment

Ingredients Treatments (metabolizable energy and protein)						
	I	II	III	IV	V	VI
Maize	44.60	41.21	38.00	50.00	47.81	44.20
Soybean meal	29.30	35.00	39.55	29.15	30.00	36.64
Gluten meal	-	-	0.50	-	3.80	2.40
Wheat bran	17	13.00	5.00	12.14	4.00	10.00
Lucerne meal	2.65	5.00	12.00	3.00	9.00	5.00
NaCl	0.41	0.40	0.40	0.40	0.40	0.40
Mineral mixture	0.25	0.25	0.25	0.25	0.25	0.25
Vitamin mixture	0.25	0.25	0.25	0.25	0.25	0.25
Anti-fungus	0.01	0.01	0.01	0.01	0.01	0.01
DL-methionine	0.10	0.10	0.10	0.10	0.10	0.10
Price, Rial · kg^−1^	9088.78	9196.41	9317.67	8890.55	9507.04	9255.80

**Table 2:** Selected ingredients and calculated analysis of used ostrich diets

	Treatments					
	I	II	III	IV	V	VI
Ingredients Dry Matter, %	83.94	86.61	84.60	85.11	85.56	88.20
Ether Extract, %	2.53	2.68	2.42	2.60	2.35	2.51
Crude fiber, %	5.44	5.14	5.80	5.46	6.59	5.68
Calcium, %	1.56	1.50	1.50	1.50	1.50	0.37
Total Phosphorus, %	1.27	0.99	1.10	0.89	0.83	0.64
Potassium, %	0.99	0.96	1.09	1.01	0.24	1.10
Sodium, mg · kg^−1^	0.19	0.18	0.19	0.19	0.19	0.18
Linoleic Acid, %	1.40	1.49	1.30	1.29	1.15	1.32
Folic acid, mg · kg^−1^	1.40	1.38	1.49	1.48	1.67	1.49
Manganese, mg · kg^−1^	419.48	411.22	415.12	403.92	406.93	403.59
Choline, g · kg^−1^	819.50	822.25	978.69	851.46	1107.49	1032.29
Lysine, %	1.02	1.01	1.16	1.06	1.29	1.22
Methionine, %	0.41	0.41	0.44	0.47	0.47	0.48
Cysteine, %	0.31	0.31	0.34	0.34	0.37	0.37
Methionine + Cysteine, %	0.72	0.72	0.77	0.81	0.84	0.85
Phenylalanine, %	0.95	0.96	1.06	1.10	1.18	1.18
Tyrosine, %	0.84	0.86	0.94	0.97	1.03	1.04
Phenylalanine + Tyrosine, %	1.79	1.83	1.99	2.08	2.21	2.22
Threonine, %	0.76	0.78	0.85	0.85	0.93	0.92
Tryptophan, %	0.33	0.32	0.37	0.36	0.43	0.39
Valine, %	0.99	1.00	1.08	1.09	1.18	1.18
Calculated analysis, %						
ME, kcal · kg^−1^	2400	2400	2400	2600	2600	2600
CP, %	20	22	24	20	22	24

During the experimental period, the feed intake (calculated as the amount of feed prepared on the first day of the week minus unconsumed feed on the last day of the week) and bodyweight gain were measured on a weekly basis using a digital balance (MDS 15000AP, Mahak Co, Tehran, Iran). Feed conversion ratio was calculated by dividing the feed intake by bodyweight gain.

## Blood samples collection and haematological analysis

At nine weeks of age, blood samples (1.5 ml) were collected from the jugular vein of one ostrich randomly selected from each replicate (three ostriches from each treatment). Blood plasma was isolated by centrifugation at 3000 rpm for 20 min at 4 °C followed by storage at − 20 °C until analysis. Glucose (mg · dl^−1^), blood urea nitrogen (mg · dl^−1^), creatinine (mg · dl^−1^), total cholesterol (mg · dl^−1^), triglycerides (mg · dl^−1^), HDL high density lipoproteins (HDL) (mg · dl^−1^), low density lipoproteins (LDL) (mg · dl^−1^), very low density lipoprotein (VLDL) (mg · dl^−^), calcium (mg · dl^−^), phosphorus (mg · dl^−^), iron (IU · dl^−1^), total protein (g · dl^−^), albumin (g · dl^−^), globulin (g · dl^−^), aspartate aminotransferase (AST; S.G.O.T.; EC 2.6.1.1; IU · l^−1^), alkaline phosphatase (ALP; EC 3.1.3.1; IU · l^−1^), and alanine aminotransferase (ALT; S.G.P.T.; EC 2.6.1.2; IU · l^−1^) were analyzed using standard commercial kits (Pars Azmoon Co., Tehran, Iran) in an auto analyzer (Hitachi 917, Roche, New York, USA).

Briefly, the levels of plasma cholesterol and triglyceride were determined using enzymatic methods (Teif Azmoon Pars, Co., Tehran, Iran), HDL and LDL cholesterol were measured directly with HDL-C and LDL-C diagnostic kits (Teif Azmoon Pars Co, Tehran, Iran). The concentration of VLDL-C was calculated by dividing plasma triglyceride by five. The LDL-C value was calculated using the formula: LDL-C = Total cholesterol – HDL-C – VLDL-C. The colorimetric determination of cholesterol in blood plasma samples involved the use of cholesterol oxidase and reaction based on the formation of a red purple quinoneimine dye, produced by oxidative condensation of a phenolic compound with 4-aminoantipyrine in the presence of hydrogen peroxide. The absorbance of the quinoneimine dye was measured spectrophotometrically; it has a direct relationship with the amount of cholesterol in the sample (Grillo et al. 1981). Plasma triglycerides were measured using a series of coupled reactions in which triglycerides are hydrolyzed to produce glycerol. The latter is converted to pyruvate and then to lactate. Decreased absorbance, measured spectrophotmetrically, is proportional to the triglyceride concentration in the sample (Schmid and Von Forstner 1986). HDL cholesterol and LDL cholesterol were measured using direct homogeneous assays. Colorimetric assays were used to determine plasma glucose using a glucose oxidase procedure. Albumin was determined based on the bromocresol green method (Doumas et al. 1971). Uric acid was determined by an enzymatic method using the uricase-TOOS method (Kayamori et al. 1997). Total protein was assayed by the Biuret method of Gornall et al. (1949). Globulin concentration is calculated by subtracting albumin concentration from total protein. Concentration of plasma alkaline phosphatase was determined enzymatically using commercial diagnostic kits (Teif Azmoon Pars, Tehran, Iran). In this procedure, alkaline phosphatase activity was determined colorimetrically by a modified method of Bessey et al. (1946). AST and ALT were determined using commercial diagnostic kits BIO-LA-TEST ALT, AST (Erba Lachema, Brno, Czech Republic). Enzymatic methods, using highly specific enzymes were developed for the determination of glucose, creatinine, calcium, phosphorus and iron. Finally, the ratios of HDL/LDL, LDL/HDL cholesterol and albumin/globulin were calculated.

## Statistical analysis

The obtained data were analyzed using a two-way analysis of variance (ANOVA) followed by Duncan’s post hoc test using a 2 × 3 factorial design with two dietary energy levels (2400 and 2600 kcal · kg^−1^ in the diet) and three dietary protein levels (20%, 22%, and 24% in the diet). Data were analyzed by SAS (2003) statistical software and GLM procedure was used. The results were considered significantly different when *P* < 0.05.

## Results

Table 3 shows feed intake of ostriches fed diets containing different levels of energy and protein from 2nd to 9th weeks of age. Mean feed intake of ostriches for all experimental periods was between 13.40 and 17.16 (kg per period). Based on these results, it was calculated that feed intake was influenced by the energy and protein levels. This parameter was higher at a lower energy level. The best (*P* ≤ 0.05) value of feed intake ratio (13.4 kg per period) was observed in ostriches fed diet contained 24% CP than those fed 20% CP (13.68 kg per period) or those fed 22% CP (14.68 kg per period) as shown in Table 3. Lower feed intake was achieved at the highest level of protein. However, increasing dietary protein resulted in decreased feed conversion (Table 4). The highest level of feed conversion was observed in birds fed treatment IV (2600 kcal · kg^−1^ dietary energy and 20% of protein). In general, the feed conversion ratio was higher with higher levels of energy (IV-VI treatments). Mean bodyweight gain level for the whole experimental period (2nd –9th week) ranged from 7.72 to 10.59 (kg per period) (Table 5). The lowest level of bodyweight gain was observed in treatment VI (2600 kcal · kg^−1^ dietary energy and 24% protein), while the highest level was in treatment I (2400 kcal · kg^−1^ dietary energy and 20% protein). Bodyweight gains were higher at a lower energy level (I-III treatments), but protein level had little impact on bodyweight gain. In general, an increase in protein level showed a depressive effect on both feed intake and bodyweight gain. The lowest levels of these two parameters were observed with diet containing 24% protein.

**Table 3:** Mean feed intake ratio (± SE) of ostrich at consecutive weeks of age fed diets containing the different levels of energy and protein from 2nd to 9th week of age, kg per period

Trait Treatment		2nd week of age	3rd week of age	4th week of age	5th week of age	6th week of age	7th week of age	8th week of age	9th week of age	First month (2nd–5th week of age)	Second month (6th–9th week of age)	Whole experiment (2nd–9th week of age)
Energy, kcal · kg^−1^ DM	2400	0.43^a^ ± 0.20	1.13^a^ ± 0.25	1.17^a^ ± 0.22	1.93^a^ ± 0.40	2.31^a^ ± 0.63	2.91^a^ ± 0.47	2.71^a^ ± 0.41	3.80^a^ ± 0.59	4.67^a^ ± 1.07	11.74^a^ ± 2.10	16.42^a^ ± 3.17
	2600	0.39^a^ ± 0.21	0.50^b^ ± 0.12	1.04^a^ ± 0.22	2.06^a^ ± 0.45	1.91^a^ ± 0.40	2.00^b^ ± 0.55	2.67^a^ ± 0.53	3.32^a^ ± 0.86	4.00^a^ ± 1.00	9.91^b^ ± 2.34	13.92^b^ ± 3.34
Protein, % in diet	20	0.48^a^ ± 0.22	0.96^a^ ± 0.36	1.23^a^ ± 0.13	1.92^a^ ± 0.37	2.23^a^ ± 0.51	2.37^a^ ± 0.84	2.43^a^ ± 0.52	3.45^a^ ± 0.55	4.60^a^ ± 1.08	10.49^a^ ± 2.42	15.09^a^ ± 3.50
	22	0.48^a^ ± 0.22	1.02^a^ ± 0.26	1.04^a^ ± 0.29	2.17^a^ ± 0.23	2.05^a^ ± 0.77	2.42^a^ ± 0.76	2.77^a^ ± 0.51	3.95^a^ ± 0.52	4.71^a^ ± 1.00	11.20^a^ ± 2.56	15.92^a^ ± 3.56
	24	0.28^a^ ± 0.05	0.47^b^ ± 0.24	1.05^a^ ± 0.21	1.89^a^ ± 0.59	2.06^a^ ± 0.39	2.57^a^ ± 0.53	2.88^a^ ± 0.25	3.28^a^ ± 1.02	3.70^a^ ± 1.09	10.79^a^ ± 2.19	14.49^a^ ± 3.28
Energy (2400)—Protein (20)		0.34^b^ ± 0.14	1.25^a^ ± 0.24	1.26^a^ ± 0.17	2.10^a^ ± 0.15	2.47^a^ ± 0.39	2.96^a^ ± 0.42	2.60^a^ ± 0.36	3.53^a^ ± 0.39	4.95^ab^ ± 0.70	11.56^a^ ± 1.56	16.51^a^ ± 2.26
Energy (2400)—Protein (22)		0.67^a^ ± 0.11	1.23^a^ ± 0.20	1.12^a^ ± 0.34	2.28^a^ ± 0.08	2.22^a^ ± 1.06	2.79^ab^ ± 0.74	2.75^a^ ± 0.72	4.10^a^ ± 0.54	5.30^a^ ± 0.73	11.86^a^ ± 3.06	17.16^a^ ± 3.79
Energy (2400)—Protein (24)		0.30^b^ ± 0.03	0.91^b^ ± 0.22	1.14^a^ ± 0.16	1.42^b^ ± 0.15	2.26^a^ ± 0.51	3.00^a^ ± 0.35	2.79^a^ ± 0.03	3.77^a^ ± 0.85	3.77^c^ ± 0.56	11.82^a^ ± 1.74	15.59^a^ ± 2.30
Energy (2600)—Protein (20)		0.62^a^ ± 0.23	0.67^bc^ ± 0.09	1.21^a^ ± 0.10	1.75^ab^ ± 0.49	2.00^a^ ± 0.58	1.79^b^ ± 0.75	2.26^a^ ± 0.68	3.38^a^ ± 0.77	4.25^abc^ ± 0.91	9.43^a^ ± 2.78	13.68^a^ ± 3.69
Energy (2600)—Protein (22)		0.29^b^ ± 0.07	0.81^bc^ ± 0.06	0.97^a^ ± 0.27	2.06^a^ ± 0.30	1.89^a^ ± 0.52	2.06^ab^ ± 0.71	2.80^a^ ± 0.14	3.80^a^ ± 0.62	4.13^bc^ ± 0.70	10.55^a^ ± 1.99	14.68^a^ ± 2.69
Energy (2600)—Protein (24)		0.26^b^ ± 0.07	0.04^c^ ± 0.55	0.96^a^ ± 0.24	2.37^a^ ± 0.52	1.86^a^ ± 0.11	2.15^ab^ ± 0.20	2.97^a^ ± 0.36	2.79^a^ ± 1.08	3.63^ab^ ± 1.38	9.77^a^ ± 11.75	13.40^a^ ± 13.13

^a,b,c^– means with different superscripts within each column for each dietary treatment division differ significantly at *P* < 0.05 or values in each groups as individual within columns with superscripts are significantly different (*P* < 0.05)

**Table 4:** Mean feed conversion ratio (± SE) of ostrich at consecutive weeks of age fed diets containing the different levels of energy and protein from 2^nd^ to 9^th^ week of age

Trait Treatment		2nd week of age	3rd week of age	4th week of age	5th week of age	6th week of age	7th week of age	8th week of age	9th week of age	First month (2nd–5th week of age)	Second month (6th–9th week of age)	Whole experiment (2nd–9th week of age)
Energy, kcal · kg^−1^ DM	2400	1.02^a^ ± 0.53	1.74^a^ ± 0.46	1.01^a^ ± 0.31	1.71^a^ ± 1.28	2.00^a^ ± 0.89	1.46^a^ ± 0.64	2.02^a^ ± 0.57	1.80^a^ ± 0.55	1.37^b^ ± 0.64	1.82^a^ ± 0.66	1.59^a^ ± 0.65
	2600	1.35^a^ ± 0.74	1.01^a^ ± 0.28	1.26^a^ ± 0.30	2.11^a^ ± 0.58	2.04^a^ ± 0.87	1.61^a^ ± 0.43	1.97^a^ ± 1.01	1.82^a^ ± 0.40	1.42^a^ ± 0.47	1.85^a^ ± 0.67	1.64^a^ ± 0.57
Protein, % in diet	20	1.19^ab^ ± 0.67	1.60^a^ ± 0.57	1.32^a^ ± 0.31	1.79^a^ ± 0.82	2.22^a^ ± 0.91	1.58^a^ ± 0.49	2.18^a^ ± 0.48	1.65^a^ ± 0.33	1.47^a^ ± 0.59	1.90^a^ ± 0.55	1.68^a^ ± 0.57
	22	1.45^a^ ± 0.71	1.74^a^ ± 0.35	0.95^b^ ± 0.33	2.28^a^ ± 1.20	1.78^a^ ± 0.81	1.58^a^ ± 0.69	1.63^a^ ± 0.60	1.92^a^ ± 0.37	1.60^a^ ± 0.64	1.72^a^ ± 0.61	1.66^a^ ± 0.62
	24	0.91^b^ ± 0.19	0.79^a^ ± 0.20	1.14^ab^ ± 0.25	1.65^a^ ± 0.64	2.06^a^ ± 0.94	1.44^a^ ± 0.47	2.18^a^ ± 1.09	1.87^a^ ± 0.67	1.12^a^ ± 0.32	1.88^a^ ± 0.79	1.50^b^ ± 0.55
Energy (2400)—Protein (20)		0.57^a^ ± 0.22	2.01^a^ ± 0.51	1.07^ab^ ± 0.17	1.54^ab^ ± 0.45	2.94^a^ ± 0.48	1.21^a^ ± 0.03	2.73^a^ ± 0.00	1.34^a^ ± 0.16	1.29^a^ ± 0.33	2.05^a^ ± 0.67	1.67^a^ ± 0.50
Energy (2400)—Protein (22)		1.59^a^ ± 0.35	1.73^ab^ ± 0.52	0.80^b^ ± 0.34	2.56^a^ ± 1.59	1.53^a^ ± 1.09	1.70^a^ ± 0.93	1.66^a^ ± 0.79	1.89^a^ ± 0.51	1.67^a^ ± 0.70	1.69^a^ ± 0.83	1.68^ab^ ± 0.76
Energy (2400)—Protein (24)		0.90^a^ ± 0.15	1.49^ab^ ± 0.30	1.17^ab^ ± 0.35	1.04^b^ ± 0.30	1.55^a^ ± 0.24	1.47^a^ ± 0.42	1.69^a^ ± 0.46	2.19^a^ ± 0.55	1.15^a^ ± 0.27	1.72^a^ ± 0.41	1.43^b^ ± 0.34
Energy (2600)—Protein (20)		1.82^a^ ± 0.29	1.19^b^ ± 0.07	1.57^a^ ± 0.20	2.05^ab^ ± 1.00	1.51^a^ ± 0.69	1.96^a^ ± 0.38	1.64^a^ ± 0.52	1.96^a^ ± 0.15	1.65^a^ ± 0.39	1.76^a^ ± 0.43	1.70^a^ ± 0.41
Energy (2600)—Protein (22)		1.31^a^ ± 1.06	1.76^ab^ ± 0.19	1.10^ab^ ± 0.29	2.01^ab^ ± 0.10	2.03^a^ ± 0.23	1.46^a^ ± 0.06	1.60^a^ ± 0.42	1.95^a^ ± 0.19	1.54^a^ ± 0.41	1.76^a^ ± 0.22	1.65^a^ ± 0.31
Energy (2600)—Protein (24)		0.92^a^ ± 0.25	0.09^b^ ± 0.01	1.11^ab^ ± 0.17	2.27^ab^ ± 0.32	2.58^a^ ± 1.01	1.42^a^ ± 0.61	2.67^a^ ± 1.33	1.55^a^ ± 0.64	1.09^a^ ± 0.18	2.05^a^ ± 0.89	1.57^ab^ ± 0.53

^a,b^– means with different superscripts within each column for each dietary treatment division differ significantly at *P* < 0.05 or values in each groups as individual within columns with superscripts are significantly different (*P* < 0.05)

**Table 5:** Mean bodyweight gain (± SE) of ostrich at consecutive weeks of age fed diets containing different levels of energy and protein from 2nd to 9th week of age, kg per period

Trait Treatment		2nd week of age	3rd week of age	4th week of age	5th week of age	6th week of age	7th week of age	8th week of age	9th week of age	First month (2nd–5th week of age)	Second month (6th–9th week of age)	Whole experiment (2nd–9th week of age)
Energy, kcal · kg^−1^ DM	2400	0.44^a^ ± 0.15	0.64^a^ ± 0.10	1.17^a^ ± 0.28	1.20^a^ ± 0.44	1.24^a^ ± 0.36	2.03^a^ ± 0.77	1.41^a^ ± 0.75	2.17^a^ ± 0.62	3.47^a^ ± 0.97	6.86^a^ ± 2.50	10.34^a^ ± 3.47
	2600	0.28^b^ ± 0.10	0.47^b^ ± 0.08	0.83^b^ ± 0.19	0.97^a^ ± 0.26	0.99^a^ ± 0.33	1.27^b^ ± 0.53	1.41^a^ ± 0.49	1.81^a^ ± 0.18	2.56^b^ ± 0.63	5.49^b^ ± 1.44	8.05^b^ ± 2.07
Protein, % in diet	20	0.46^a^ ± 0.18	0.59^a^ ± 0.09	0.97^a^ ± 0.33	1.10^a^ ± 0.42	1.08^a^ ± 0.29	1.67^a^ ± 0.90	1.16^a^ ± 0.81	2.17^a^ ± 0.63	3.13^a^ ± 1.02	6.08^a^ ± 2.63	9.21^a^ ± 3.65
	22	0.32^b^ ± 0.15	0.58^a^ ± 0.16	1.13^a^ ± 0.36	0.95^a^ ± 0.40	1.19^a^ ± 0.35	1.52^a^ ± 0.84	1.70^a^ ± 0.45	2.05^a^ ± 0.37	2.99^a^ ± 1.07	6.46^a^ ± 2.01	9.46^a^ ± 3.08
	24	0.30^b^ ± 0.07	0.51^a^ ± 0.11	0.91^a^ ± 0.12	1.20^a^ ± 0.30	1.08^a^ ± 0.46	1.77^a^ ± 0.66	1.38^a^ ± 0.42	1.75^a^ ± 0.40	2.93^a^ ± 0.6	5.99^a^ ± 1.94	8.92^a^ ± 2.54
Energy (2400)—Protein (20)		0.59^a^ ± 0.17	0.62^a^ ± 0.13	1.17^ab^ ± 0.36	1.36^a^ ± 0.26	0.84^c^ ± 0.11	2.43^a^ ± 0.35	0.95^a^ ± 1.15	2.63^a^ ± 0.68	3.74^a^ ± 0.92	6.85^a^ ± 2.29	10.59^ab^ ± 3.21
Energy (2400)—Protein (22)		0.42^ab^ ± 0.11	0.71^a^ ± 0.11	1.39^a^ ± 0.14	0.89^a^ ± 0.58	1.45^a^ ± 0.27	1.64^ab^ ± 1.18	1.65^a^ ± 0.58	2.16^a^ ± 0.49	3.41^ab^ ± 0.94	6.90^a^ ± 2.52	10.31^a^ ± 3.46
Energy (2400)—Protein (24)		0.33^b^ ± 0.02	0.61^ab^ ± 0.04	0.97^b^ ± 0.15	1.36^a^ ± 0.38	1.45^a^ ± 0.29	2.03^ab^ ± 0.63	1.65^a^ ± 0.36	1.72^a^ ± 0.62	3.27^ab^ ± 0.59	6.85^a^ ± 1.90	10.12^a^ ± 2.49
Energy (2600)—Protein (20)		0.34^b^ ± 0.08	0.56^abc^ ± 0.04	0.77^b^ ± 0.14	0.85^a^ ± 0.43	1.32^ab^ ± 0.20	0.91^b^ ± 0.42	1.37^a^ ± 0.40	1.72^a^ ± 0.25	2.52^b^ ± 0.69	5.32^a^ ± 1.27	7.84^b^ ± 1.96
Energy (2600)—Protein (22)		0.22^b^ ± 0.12	0.46^bc^ ± 0.08	0.88^b^ ± 0.32	1.02^a^ ± 0.20	0.93^bc^ ± 0.22	1.41^ab^ ± 0.08	1.75^a^ ± 0.36	1.94^a^ ± 0.12	2.58^b^ ± 0.72	6.03^a^ ± 0.78	8.61^ab^ ± 1.50
Energy (2600)—Protein (24)		0.28^b^ ± 0.11	0.41^c^ ± 0.03	0.86^b^ ± 0.09	1.04^a^ ± 0.09	0.72^c^ ± 0.26	1.51^ab^ ± 0.71	1.11^a^ ± 0.32	1.79^a^ ± 0.11	2.59^b^ ± 0.32	5.13^a^ ± 1.40	7.72^b^ ± 1.72

^a,b,c^– means with different superscripts within each column for each dietary treatment division differ significantly at *P* < 0.05 or values in each groups as individual within columns with superscripts are significantly different (*P* < 0.05)

The results of the blood chemistry parameters are presented in Table 6. The lowest levels of glucose and triglycerides were in treatment I (2400 kcal · kg^−1^ dietary energy and 20% protein). Total cholesterol was between 115.67 and 218.67 mg · dl^−1^. The lowest levels of total cholesterol, LDL cholesterol and HDL cholesterol were observed in treatment V (2600 kcal · kg^−1^ dietary energy and 22% of protein). HDL/LDL cholesterol ratio was the lowest at 2600 kcal · kg^−1^ dietary energy level (IV–VI treatments). The alkaline phosphatase level was 1116.70 IU · l^−1^ in treatment II (2400 kcal · kg^−1^ dietary energy and 22% of protein) and 1957.30 IU · l^−1^ in treatment VI (2600 kcal · kg^−1^ dietary energy and 24% protein). Blood urea nitrogen and creatinine were similar in all six treatments. The total protein level ranged from 2.20 g · dl^−1^ in treatment V (2600 kcal · kg^−1^ dietary energy and 22% protein) to 2.66 g · dl^−1^ in treatment III (2400 kcal · kg^−1^ dietary energy and 24% protein) (Table 6). The highest level of albumin was found in treatment III (1.60 g · dl^−1^), whereas the highest level of globulin was observed in treatment II (2400 kcal · kg^−1^ dietary energy and 22% protein) – 1.16 g · dl^−1^.

**Table 6:** Mean blood chemistry parameters (± SE) of ostrich at 9th week of age fed diets containing different levels of energy and protein from 2nd to 9th week of age

Trait Treatment		Glucose, mg · dl^−1^	Blood urea nitrogen, mg · dl^−1^	Creatinine, mg · dl^−1^	Total cholesterol, mg · dl^−1^	Triglycerides, mg · dl^−1^	HDL Cholesterol (High Density Lipoproteins), mg · dl^−1^	LDL Cholesterol (Low Density Lipoproteins), mg · dl^−1^	Aspartat Amino Transferase (AST) (S.G.O.T) (EC 2.6.1.1), IU · l^−1^	Alkaline Phosphatase (ALP) (EC 3.13.1), IU · l^−1^	Alanin Amino Transferase (ALT) (S.G.P.T) (EC 2.6.1.2), IU · l^−1^
Energy, kcal · kg^−1^ DM	2400	219.44^a^ ± 27.73	1.00^a^ ± 0.00	0.22^a^ ± 0.00	187.22^a^ ± 39.79	113.67^a^ ± 45.00	43.33^a^ ± 14.50	121.22^a^ ± 34.67	471.11^b^ ± 77.87	1272.20^a^ ± 226.53	15.77^a^ ± 8.59
	2600	238.22^a^ ± 36.27	1.00^a^ ± 0.00	0.22^a^ ± 0.02	142.89^b^ ± 42.94	128.33^a^ ± 45.46	24.77^b^ ± 11.76	92.33^a^ ± 42.10	650.22^a^ ± 200.32	1535.10^a^ ± 558.60	21.22^a^ ± 5.93
Protein, % in diet	20	222.17^a^ ± 27.52	1.00^a^ ± 0.00	0.22^a^ ± 0.01	166.33^a^ ± 37.08	125.33^a^ ± 58.59	36.50^a^ ± 12.92	104.67^a^ ± 44.61	538.33^b^ ± 101.47	1412.30^a^ ± 326.30	15.83^a^ ± 1.47
	22	245.00^a^ ± 47.79	1.00^a^ ± 0.00	0.22^a^ ± 0.01	167.17^a^ ± 60.07	129.67^a^ ± 50.52	35.16^a^ ± 24.06	106.00^a^ ± 44.14	676.00^a^ ± 253.26	1205.00^a^ ± 200.67	23.00^a^ ± 9.89
	24	219.33^a^ ± 11.69	1.00^a^ ± 0.00	0.22^a^ ± 0.03	161.67^a^ ± 47.41	108.00^a^ ± 20.96	30.50^a^ ± 9.91	109.67^a^ ± 39.45	467.67^b^ ± 37.46	1593.70^a^ ± 637.23	16.66^a^ ± 8.01
Energy (2400)—Protein (20)		198.67^a^ ± 6.42	1.00^a^ ± 0.00	0.22^a^ ± 0.00	160.33^ab^ ± 12.42	89.67^a^ ± 30.61	40.33^ab^ ± 13.31	102.00^a^ ± 28.82	470.00^b^ ± 55.67	1470.00^ab^ ± 187.34	15.66^ab^ ± 1.15
Energy (2400)—Protein (22)		236.33^a^ ± 42.19	1.00^a^ ± 0.00	0.23^a^ ± 0.00	218.67^ab^ ± 10.01	146.67^a^ ± 62.14	54.66^a^ ± 14.36	134.67^a^ ± 31.53	480.00^b^ ± 141.77	1116.70^b^ ± 225.46	19.33^ab^ ± 12.66
Energy (2400)—Protein (24)		223.33^a^ ± 12.50	1.00^a^ ± 0.00	0.22^a^ ± 0.01	182.67^ab^ ± 59.01	104.67^a^ ± 26.10	35.00^abc^ ± 12.16	127.00^a^ ± 45.92	463.33^b^ ± 29.14	1230.00^ab^ ± 147.30	12.33^b^ ± 9.86
Energy (2600)—Protein (20)		245.67^a^ ± 14.01	1.00^a^ ± 0.00	0.21^a^ ± 0.01	172.33^ab^ ± 56.35	161.00^a^ ± 61.87	32.66^abc^ ± 14.01	107.33^a^ ± 64.22	606.67^b^ ± 92.91	1354.70^ab^ ± 470.21	16.00^ab^ ± 2.00
Energy (2600)—Protein (22)		253.67^a^ ± 60.86	1.00^a^ ± 0.00	0.22^a^ ± 0.01	115.67^b^ ± 31.08	112.67^a^ ± 40.64	15.66^c^ ± 10.01	77.33^a^ ± 37.58	872.00^a^ ± 158.15	1293.30^ab^ ± 162.58	26.66^a^ ± 6.6
Energy (2600)—Protein (24)		215.33^a^ ± 11.71	1.00^a^ ± 0.00	0.22^a^ ± 0.04	140.67^b^ ± 28.53	111.33^a^ ± 19.60	26.00^bc^ ± 6.08	92.33^a^ ± 29.68	472.00^b^ ± 51.02	1957.30^a^ ± 772.46	21.00^ab^ ± 2.64

^a,b,c^– means with different superscripts within each column for each dietary treatment division differ significantly at *P* < 0.05 or values in each groups as individual within columns with superscripts are significantly different (*P* < 0.05)

In general, most of the studied blood chemistry parameters were lower at low energy level (I-III treatments). Total cholesterol, HDL, LDL, protein, albumin and globulin were higher at lower energy level. The energy and protein levels had no significant effect (*P* < 0.05) on blood urea nitrogen and creatinine. There were no significant differences in levels of minerals, e.g., calcium, phosphorus and iron.

## Discussion

A well-balanced diet allows good absorption of minerals and vitamins, which leads to the best development parameters of an ostrich, especially its growth and reproduction (Cooper and Horbańczuk 2004). The most sensitive to feeding are small birds, up to three months of age. Hence, proper nutrition of ostriches is very important (Cooper 2000). Published articles on ostrich nutrition are increasingly used by breeders of these birds. In the present study we monitored the dynamics of feed and body parameters. We also defined how different levels of protein and energy affect feed intake, bodyweight and blood parameters in ostriches from the 2nd to 9th weeks of age.

The statistical analysis of the overall study showed a significant effect of diet on feed intake, feed conversion ratio and bodyweight gain (*p* < 0.05). Brand et al. (2010) showed that the different energy level in the feed had no effect on feed intake and reproductive parameters in the ostrich. In turn, Dairo et al. (2010) reported that low energy and protein level in the diet of broiler chickens, decreases feed intake. Nevertheless, the final live weight of broiler chickens was similar to that of birds fed a normal diet. These studies show that birds can be fed a diet of low energy and protein content without compromising their final weight. In another study, Brand et al. (2014) obtained the best results with medium energy level. Mahrose et al. (2015) indicated that different levels of protein in the diet had little effect on feed intake, body weight and weight gain in young ostriches. These authors found that birds at the age of 2–9 weeks did well with feed of a lower protein level (around 18%), which was later confirmed by Mahrose et al. (2019).

According to the results of the present study, it can be deduced that the mean bodyweight gain was higher in the case of lower energy level (I-III treatment) as has been reported by Tasirnafas et al. (2015). These authors also studied feed intake and feed conversion. The mean feed intake in all eight treatments was between 27.71 and 38.15 kg per ostrich. The energy levels of dietary treatments had no significant effect (*P* < 0.05) on feed intake.

The highest level of feed conversion ratio was found in ostriches fed a diet containing higher level of energy (treatment VIII – 2700 kcal/kg dietary energy). Meanwhile, statistical differences between studied treatments for this trait were not significant (*P* < 0.05). Glatz et al. (2008) reported that ostriches fed diet of low energy and low protein (10.0 MJ per kg and 126 g per kg) had better growth performance compared with those fed high energy and high protein (12.5 MJ per kg and 143 g per kg) diet. In our study, bodyweight gain was higher at a low energy level. Protein level had little impact on bodyweight gain. In fact, an increase in protein levels showed a depressive effect on feed intake and bodyweight gain.

Blood parameters are influenced by many factors including age, condition, sex, nutrition and illness. The results of blood test can testify to the condition of a given individual and on their basis can recognize many diseases.

One of the studied blood parameters was glucose. It’s content in the blood of healthy birds is about 200–500 mg per dl, which is higher than in mammals. This difference is due to glucose homeostasis in mammals, where the main role is played by insulin. In birds however glucagon plays this role. Glucose concentration in our study was around 200 mg per dl. In previous studies, the highest level of glucose belonged to diet containing higher level of energy (Tasirnafas et al. 2014). Our results also reinforced this observation.

The concentration of blood urea in the blood of fasting birds ranges from 0 to 10 mg per dl. The urea values in our study were within the normal limit for birds in general but closer to the lower limit (1.0 mg per dl). Durgan et al. (2005), on the other hand, obtained a value of more than 4 mg per dl.

It is well known that the serum cholesterol level of birds increases in relation to higher fat content and lower protein content in the diet. Our studies have confirmed this dependence. Tasirnafas et al. (2015) also reported similar results; total cholesterol concentration was higher for ostriches fed lower energy diet. Cholesterol concentrations in our study were higher than reported in previous studies (Durgun et al. 2005; Samour et al. 2011; Khodaei Motlagh and Masoumi 2016).

Our results indicate that triglycerides can also be influenced by diet. The values of this parameter ranged from 89.67 mg per dl to 161.00 mg per dl and were similar to previously studies of Durgun et al. (2005) and Khodaei Motlagh and Masoumi (2016).

Serum creatinine levels were similar to previously reported values in ostrich (Durgun et al. 2005). Plasma ALT activity is a specific nor a sensitive test of hepatocellular disease in bird and has no advantages as compared with AST as a test for hepatocellular disease (Thrall et al. 2004). Plasma alkaline phosphatase (ALP) in this study was much higher than values published by Hassim et al. (2006). It should be remembered that plasma ALP activity in birds primarily results from osteo blastic activity. ALP activity in birds is the result of osteoblastic activity; hence it is not useful for the detection of hepatobiliary disease (Thrall et al. 2004). Aspartate aminotransferase (AST) activity was at much higher level than previously reported Hassim et al. (2006) ad Khodaei Motlagh and Masoumi (2016). However, Durgun et al. (2005) and Samour et al. (2011) reported similar values of AST enzyme activity to ours. Alanine aminotransferase (ALT) activity has a wide range in several species of birds (Thrall et al. 2004). The results from this study were less than those reported by Khodaei Motlagh and Masoumi (2016) but more than reported by Durgun et al. (2005) and Hassim et al. (2006).

The level of total proteins in birds is less than in mammals (3 to 5 g per dl for most birds; Coleman et al. 1988). Ostrich total protein has been reported previously as within these normal values (Khodaei Motlagh and Masoumi 2016) and the current study results are not consistent with those previously reported. They were below normal and ranged from 2.20 g per dl to 2.66 g per dl.

All differences observed in presented studies may have been due to other causes including management, environmental temperature at the time of sampling, sample storage temperature, and the time-lapse between collection and processing.

## Conclusions

In conclusion, our results provide quantitative information on the impact of dietary energy and protein on feed intake, feed conversion, bodyweight gain and selected blood chemistry parameters in ostriches. An increase in the level of protein showed depressive effect on feed intake and bodyweight gain. It was concluded that selected blood chemistry parameter values were influenced by the energy and protein level. The data obtained in the study could be useful to establish some baseline values for blood parameters in ostriches, which could be used as a reference for detecting metabolic and nutritional disorders. These types of studies are needed to confirm that performance of ostrich can be enhanced without too much cost.
